# Neutrophil-lymphocyte and platelet-lymphocyte ratios as systemic inflammatory biomarkers for atopic dermatitis in US adults: a cross-sectional NHANES study revealing subgroup heterogeneity

**DOI:** 10.3389/fimmu.2025.1585451

**Published:** 2025-06-18

**Authors:** Xuanlin Chen, Xiangyu Yang, Min Zhang, Yirui Zhao, Shuping Guo

**Affiliations:** Department of Dermatology, The First Affiliated Hospital of Shanxi Medical University, Taiyuan, Shanxi, China

**Keywords:** atopic dermatitis, NHANES, neutrophil-to-lymphocyte ratio, platelet-to-lymphocyte ratio, systemic inflammation, biomarker

## Abstract

**Background:**

Neutrophil-to-lymphocyte ratio (NLR) and platelet-to-lymphocyte ratio (PLR) are systemic inflammation markers, but their association with adult atopic dermatitis (AD) remains underexplored.

**Methods:**

This cross-sectional study analyzed 2001–2006 NHANES data from 10,890 US adults. AD was defined by self-reported physician diagnosis. Cutoffs for NLR (1. 81×10^9^/L) and PLR (136. 13×10^9^/L) were determined via ROC analysis. Multivariable models adjusted for sociodemographic and clinical covariates.

**Results:**

Elevated NLR (≥1. 81×10^9^/L) and PLR (≥136. 13×10^9^/L) were independently associated with higher AD prevalence after full adjustment (NLR: OR=1. 23, 95%CI:1. 08–1. 40; PLR: OR=1. 24, 95%CI:1. 10–1. 41). Subgroup analyses revealed stronger associations in males, normal-BMI individuals, and asthmatics (PLR: OR=1. 84), but inverse correlations in nonsmokers (NLR: OR=0. 33; PLR: OR=0. 34). Significant interactions occurred with BMI and asthma (PLR-interaction P=0. 0077).

**Conclusion:**

NLR and PLR are accessible systemic inflammatory biomarkers for AD, with subgroup heterogeneity suggesting roles for lymphocyte depletion (skin homing), neutrophilic (Th17), and platelet-mediated (Th2) inflammation pathways.

## 1 Introduction

Atopic dermatitis (AD) is a prevalent, recurring inflammatory skin disease characterised by dry skin and unrelenting itching. It can be found in all age groups, with a global prevalence ranging from 2. 7%–20. 1% in children (1) and 2. 1%–4. 9% in adults (2). The prevalence is increasing annually (3). The etiology and pathogenesis of AD are believed to be the result of multiple factors, including genetics, immune dysregulation, and skin barrier dysfunction, in which lymphocytes play a key role (4). Lymphocyte skin homing has been described as a major feature of AD (5, 6), and lymphocytopenia has been observed in severe cases of AD (7). Consequently, further exploration into the association between lymphocyte-related factors and AD may facilitate more precise diagnosis and prognosis.

The neutrophil-lymphocyte ratio (NLR) and the platelet-lymphocyte ratio (PLR) are two easily measurable indicators of systemic inflammation, both of which have lymphocyte correlates. These indicators have been reported to be of clinical value in acute kidney injury (8), myocardial infarction (9), systemic lupus erythematosus (10), and rheumatoid arthritis (11), and are associated with prognosis in malignancies (12). In patients diagnosed with psoriasis, a positive correlation has been identified between NLR and psoriasis area and severity index (PASI) scores (13). Additionally, PLR has been observed to be associated with the response to TNF-α inhibitor therapy in patients with psoriasis (14). However, the impact of NLR and PLR on atopic dermatitis in US adults remains incompletely understood. Whilst prior research has indicated an association between NLR/PLR and the severity of AD in pediatric cohorts (15, 16) or in smaller samples (17), this study constitutes the first nationally representative analysis of the potential association between NLR and PLR with atopic dermatitis in US adults, with novel focus on subgroup heterogeneity driven by sex, body mass index (BMI), asthma, and smoking status.

## 2 Methods

### 2.1 Data sources

The National Health and Nutrition Examination Survey (NHANES), a biennial survey conducted by the National Center for Health Statistics (NCHS), provides cross-sectional data representing the nutritional and health status of the general population throughout the United States. NHANES uses stratified multistage probability sampling to represent the non-institutionalized US population. Survey weights were applied to ensure national generalizability. Due to the use of publicly available data, this cross-sectional study was considered exempt from ethical review and informed consent.

### 2.2 Study design and population

**Figure 1:**
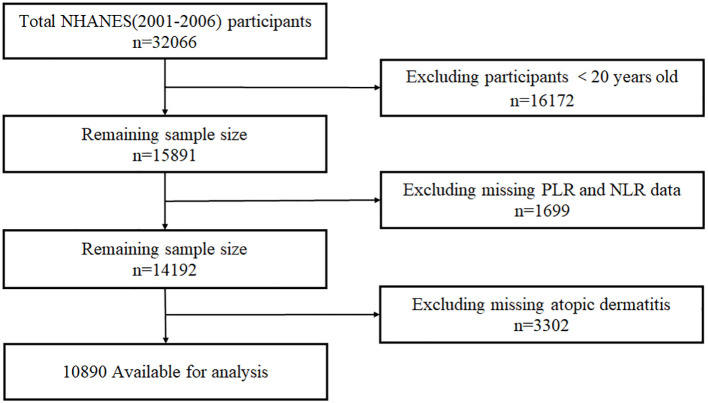
The flow diagram of the study participants, from NHANE 2001–2006.

The NHANES dataset from three consecutive cycles from 2001 to 2006 was utilized for this study, and 15,891 adults over the age of 20 were selected, excluding 1,699 participants due to missing NLR/PLR data (platelet counts, neutrophils, or lymphocytes) and 3,302 participants with missing data on atopic dermatitis. The final analysis included 10,890 eligible participants, Participant selection shown in **Figure 1**.

### 2.3 Assessment of NLR/PLR and atopic dermatitis

AD was defined as answering in the affirmative to the question, “Have you been told by a physician that you have eczema?” (18). NLR and PLR were calculated from the neutrophil, platelet counts, and lymphocyte in the peripheral blood, defined as neutrophil/lymphocyte, and platelet count/lymphocyte, respectively. The data were derived from participants’ initial routine blood tests, and the laboratory methodology for routine blood tests can be found on the NHANES website.

### 2.4 Covariates

The following covariates were selected for inclusion in the study: sex, age, race, education, household income, marriage, smoking (19), asthma (20), vitamin D (21), and BMI. Race was categorized into five groups: Mexican American, other Hispanic, non-Hispanic white, non-Hispanic black, and other race. Household income was categorized into three groups based on the household poverty-to-income ratio (22): low income (≤1. 3), middle income (>1. 3 to 3. 5), and high income (>3. 5). Education was categorized into three levels: high school or below, some college, and college graduate or above. Smoking was categorized into smokers and non-smokers based on the question “Smoked at least 100 cigarettes in your lifetime”. Individuals who responded in the affirmative to the inquiry regarding physician-diagnosed asthma were designated as asthmatics. Subjects were stratified into three categories based on their BMI: normal weight (BMI <25 kg/m²), overweight (25–29. 9 kg/m²), and obese (BMI ≥30 kg/m²).

### 2.5 Statistical analysis

In this study, we performed a receiver operating characteristic (ROC) analysis of PLR and NLR to determine the optimal cutoff values of 136. 13×10^9^/L and 1. 81×10^9^/L, respectively. We then categorized PLR and NLR into high PLR, low PLR, high NLR, and low NLR groups based on the cutoff values. To ascertain the presence of multicollinearity, we employed the variance inflation factor (VIF) method, a statistical technique that quantifies the correlation between independent variables. The VIF method was used to identify potential multicollinearity if the VIF value was 5 or higher. Model 1 adjusted for sociodemographic variables (age, sex, race, education level, household income, and marital status), and model 2 was a fully adjusted model that included sociodemographic variables, asthma, smoking, vitamin D, and BMI. In subgroup analyses of the associations of NLR and PLR with AD, sex-, smoking-, BMI-, marital-, and asthma-stratified variables were used, and interaction tests were performed with this as effect modifiers. Continuous variables are presented as median (interquartile range) and compared via Kruskal-Wallis test. Categorical variables were described by the number of instances and percentages and were compared using the chi-square test.

Since the maximum percentage of missing data for covariates was 5%, we used Python to interpolate the missing data using the random forest method. All statistical analyses were carried out using EmpowerStats software (Version 2. 0, X&Y Solutions, Inc., Boston, MA; http://www.empowerstats.com), and a significance level of *P* < 0. 05 was considered statistically significant for the results.

## 3 Results

The study’s sample population included a total of 10,890 participants. As indicated in **Table 1**, 9,768 participants were not diagnosed with AD, while 1,122 participants were diagnosed with AD. In both groups, statistically significant differences were observed in gender, race, education level, smoking, asthma, BMI subgroups, NLR subgroups, PLR subgroups, and household income (all *P* < 0. 05). Furthermore, the prevalence of atopic dermatitis was significantly higher in females, non-Hispanic whites, those with ≤high school education, and higher-income individuals (all *P* < 0. 05).

**Table 1:** Characteristics of participants in the NHANES 2001–2006 cycles.

Characteristic	Participants	Without AD ^a^	With AD	*P* value
	Total (n=10890)	(n=9768)	(n=1122)	
Age, median (IQR)	41.00 (29.00-52.00)	41.00 (29.00-52.00)	41.00 (30.00-51.00)	0.312
Vitamin D, median (IQR)	56.30 (42.00-71.30)	56.30 (42.00-71.30)	58.10 (44.40-73.60)	0.002
Sex				<0.001
male	5186 (47.62%)	4710 (48.22%)	476 (42.42%)	
female	5704 (52.38%)	5058 (51.78%)	646 (57.58%)	
Race and ethnicity ^b^				<0.001
Mexican American	2273 (20.87%)	2142 (21.93%)	131 (11.68%)	
Other Hispanic	410 (3.76%)	368 (3.77%)	42 (3.74%)	
Non-Hispanic White	5413 (49.71%)	4711 (48.23%)	702 (62.57%)	
Non-Hispanic Black	2345 (21.53%)	2149 (22.00%)	196 (17.47%)	
Other Race	449 (4.12%)	398 (4.07%)	51 (4.55%)	
Educational level				<0.001
High school or less	5440 (49.95%)	5016 (51.35%)	424 (37.79%)	
Some College	3209 (29.47%)	2821 (28.88%)	388 (34.58%)	
College Graduate or above	2241 (20.58%)	1931 (19.77%)	310 (27.63%)	
Marital status				0.413
Married	7836 (71.96%)	7017 (71.84%)	819 (72.99%)	
Never married	3054 (28.04%)	2751 (28.16%)	303 (27.01%)	
Smoke ^c^				0.017
yes	5135 (47.15%)	4568 (46.76%)	567 (50.53%)	
no	5755 (52.85%)	5200 (53.24%)	555 (49.47%)	
Asthma				<0.001
yes	1361 (12.50%)	1169 (11.97%)	192 (17.11%)	
no	9529 (87.50%)	8599 (88.03%)	930 (82.89%)	
BMI (kg/m²)				0.006
<25	3410 (31.31%)	3035 (31.07%)	375 (33.42%)	
≥25, <30	3879 (35.62%)	3528 (36.12%)	351 (31.28%)	
≥30	3601 (33.07%)	3205 (32.81%)	396 (35.29%)	
Family income ^d^				<0.001
Low	2776 (25.49%)	2542 (26.02%)	234 (20.86%)	
Medium	4457 (40.93%)	4051 (41.47%)	406 (36.19%)	
High	3657 (33.58%)	3175 (32.50%)	482 (42.96%	
PLR (10^9^/L)				<0.001
<136.13	5989 (55.00%)	5432 (55.61%)	557 (49.64%)	
≥136.13	4901 (45.00%)	4336 (44.39%)	565 (50.36%)	
NLR (10^9^/L)				<0.001
<1.81	4447 (40.84%	4055 (41.51%)	392 (34.94%)	
≥1.81	6443 (59.16%)	5713 (58.49%)	730 (65.06%)	

NHANES, National Health and Nutrition Examination Survey; AD, atopic dermatitis; BMI, body mass index (calculated as kilograms divided by meters squared); NLR, Neutrophil-to-lymphocyte ratio; PLR, platelet-to-lymphocyte ratio; IQR, interquartile range.

Data are presented as unweighted number (weighted percentage) unless otherwise indicated.

a Atopic dermatitis was defined as answering in the affirmative to the question, "Have you been told by a physician that you have eczema?"

b Race and ethnicity were self-reported.

c Smoking was categorized into smokers and non-smokers based on the question "Smoked at least 100 cigarettes in your lifetime"

d Family income was categorized into three groups based on the household poverty-to-income ratio (19): low income (≤1.3), middle income (>1.3 to 3.5), and high income (>3.5).

**Figure 2:**
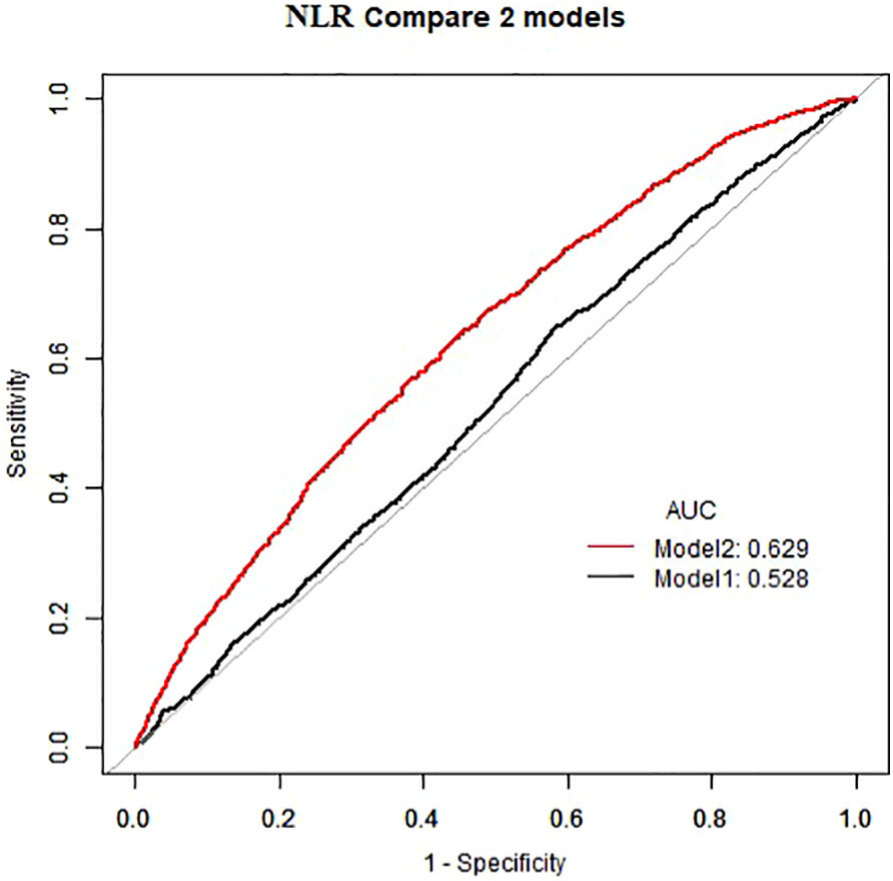
Comparing NLR AUC for crude model and fully adjusted models.

**Figure 3:**
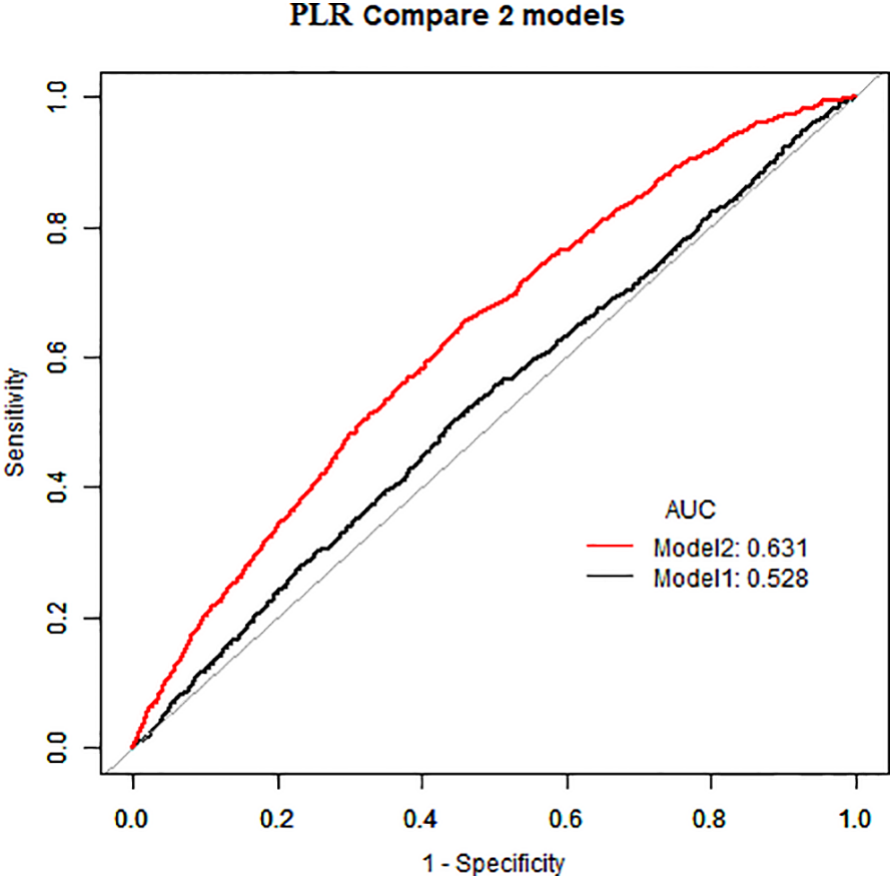
Comparing PLR AUC for crude model and fully adjusted models.

As demonstrated in **Table 2**, in the absence of adjustment for covariates (Crude model), high NLR (≥1. 81×10^9^/L) and high PLR (≥136. 13×10^9^/L) exhibited a positive correlation with the prevalence of atopic dermatitis among US adults (OR=1. 32, 95%CI:1. 16–1. 50) (OR=1. 27, 95%CI:1. 12–1. 44). This positive association remained consistent after adjusting for all potential confounders (Model 2) (OR=1. 23, 95%CI:1. 08–1. 40) (OR=1. 24, 95%CI:1. 10–1. 41). The area under the curve (AUC) values of the fully adjusted model for NLR and PLR were 0. 629 and 0. 631, respectively, both significantly higher than the crude model (**Figures 2**, **3**), the differences in these values were found to be statistically significant (*P*<0. 001). This outcome indicates that the fully adjusted model constructed in this study exhibits superior discrimination and performance, surpassing the capabilities of the univariate regression model in terms of differentiation and accuracy.

**Table 2:** Association of NLR and PLR with AD among participants in the NHANES 2001–2006 cycles.

Model	NLR (10^9^/L)		*P* value	PLR (10^9^/L)		*P* value
	<1.81	≥1.81		<136.13	≥136.13	
Crude model ^a^ (OR, 95% CI)	Ref	1.32 (1.16, 1.50)	<0.0001	Ref	1.27 (1.12, 1.44)	0.0001
Model 1 ^b^ (OR, 95% CI)	Ref	1.23 (1.08, 1.41)	0.0022	Ref	1.22 (1.07, 1.38)	0.0022
Model 2 ^c^ (OR, 95% CI)	Ref	1.23 (1.08, 1.40)	0.0024	Ref	1.24 (1.10, 1.41)	0.0008

NHANES, National Health and Nutrition Examination Survey; AD, atopic dermatitis; CI, confidence interval; OR, odds ratio; NLR, Neutrophil-to-lymphocyte ratio; PLR, platelet-to-lymphocyte ratio.

a Crude model: adjusted for none.

b Model 1 adjusted for sociodemographic variables (age, sex, race, education level, household income, and marital status).

c Model 2 was a fully adjusted model that included sociodemographic variables, asthma, smoking, vitamin D, and BMI.

Spearman correlation analysis revealed significant associations between atopic dermatitis (AD) status and hematological parameters (**Table 3**). Lymphocyte count demonstrated a modest but statistically significant negative correlation with AD status (ρ = -0. 042, *P* = 0. 016), consistent with lymphocyte trafficking to inflamed skin sites. While neutrophil (ρ = 0. 022, *P* = 0. 070) and platelet counts (ρ = 0. 023, *P* = 0. 065) showed positive trends that approached significance, the composite inflammatory ratios exhibited stronger associations: NLR correlated positively with AD status (ρ = 0. 045, *P* = 0. 010), as did PLR (ρ = 0. 050, *P* = 0. 005). These findings indicate that the reduction in peripheral lymphocytes amplifies the inflammatory signal captured by NLR and PLR beyond what is detectable through individual cell counts alone.

**Table 3:** Spearman correlations between AD status and hematological parameters.

Parameter	ρ	*P* value	95% CI
Lymphocyte count	-0.042	0.016	(-0.076, -0.008)
Neutrophil count	0.022	0.070	(-0.002, 0.046)
Platelet count	0.023	0.065	(-0.001, 0.047)
NLR	0.045	0.010	(0.011, 0.079)
PLR	0.050	0.005	(0.015, 0.085)

AD, atopic dermatitis; CI, confidence interval; NLR, Neutrophil-to-lymphocyte ratio; PLR, platelet-to-lymphocyte ratio.

**Figure 4:**
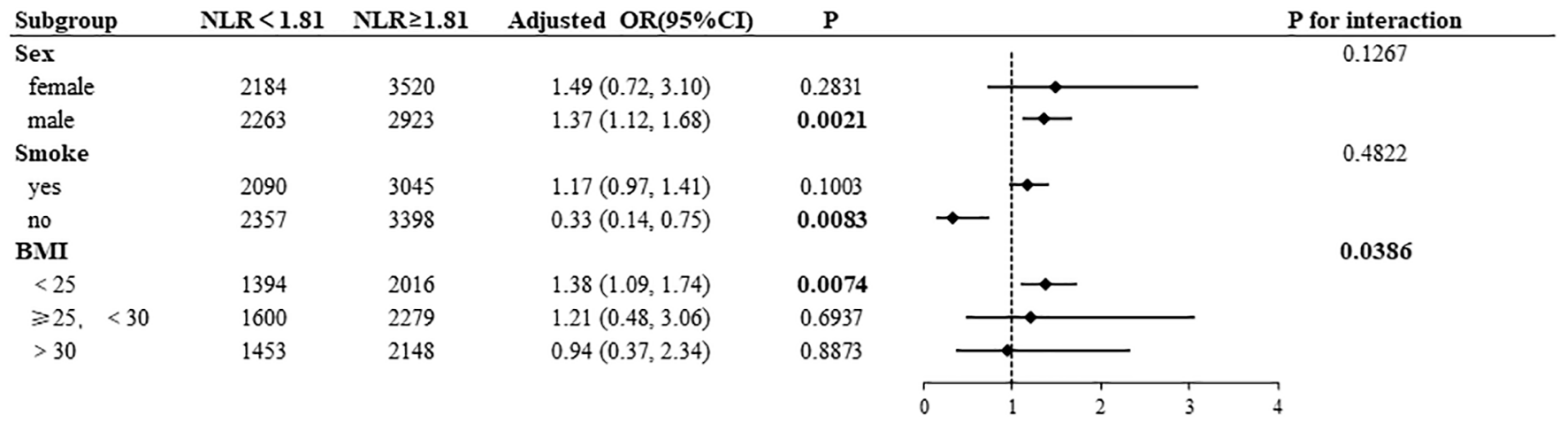
Subgroup analysis of NLR and AD association.

**Figure 5:**
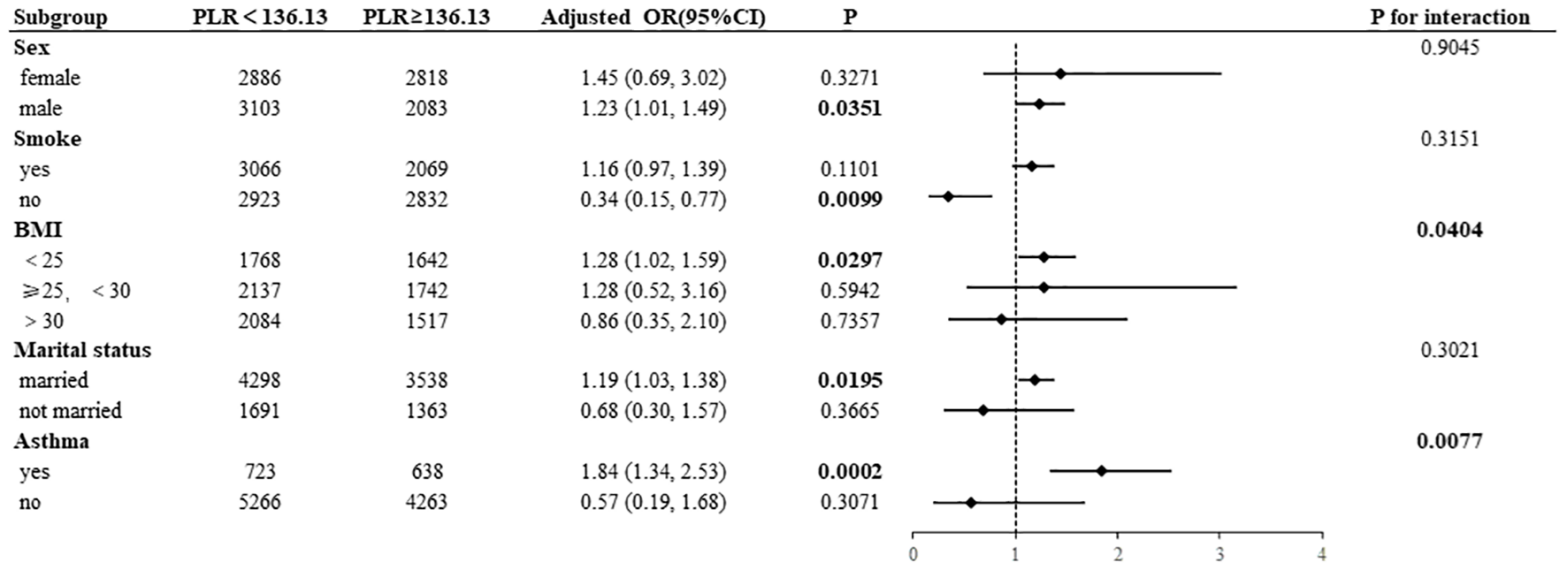
Subgroup analysis of PLR and AD association.

Subgroup analyses were conducted to examine the associations of NLR and PLR with AD, the results of which are presented in **Figures 4**, **5**. High NLR (≥1. 81×10^9^/L) was found to be negatively associated with the prevalence of AD in nonsmoking participants (OR= 0. 33, 95% CI:0. 14–0. 75). Conversely, the prevalence of AD was positively associated with high NLR in male participants (OR=1. 37, 95% CI:1. 12–1. 68) and in BMI <25 kg/m² (OR=1. 38, 95% CI:1. 09–1. 74). A statistically significant interaction was observed between NLR and AD in the BMI subgroup (*P* for interaction=0. 0386). In a manner analogous to the association between NLR and AD, high PLR (≥136. 13×10^9^/L) exhibited a negative correlation with the prevalence of AD in nonsmoking participants (OR=0. 34, 95% CI:0. 15–0. 77). Conversely, high PLR demonstrated a positive correlation with the prevalence of AD in male participants (OR=1. 23, 95% CI:1. 01–1. 49) and in those with a BMI <25 kg/m² (OR=1. 28, 95% CI:1. 02–1. 59), had asthma (OR=1. 84, 95% CI:1. 34-2. 53), and were married (OR=1. 19, 95% CI:1. 03–1. 38). A statistically significant interaction was observed between PLR and AD in the BMI subgroup (*P* for interaction=0. 0404) and the asthma subgroup (*P* for interaction=0. 0077).

## 4 Discussion

This study provides the first evidence of significant associations between NLR and PLR with the risk of developing AD in adults, based on a large national sample of US data. The findings of this study, when assessed in a fully adjusted model, indicate that elevated NLR (≥1. 81×10^9^/L) and PLR (≥136. 13×10^9^/L) are associated with a 23% and 24% increase in the prevalence of AD, respectively. These results align with the observations reported by Japanese scholars Inokuchi et al., who noted a correlation between NLR and the severity of the disease in a smaller sample size study (23). Notably, subgroup analyses revealed significant heterogeneity: stronger positive associations were observed in males, normal-BMI individuals, and asthmatics, whereas an inverse association was observed in nonsmokers. This discrepancy suggests that the association of inflammatory markers with atopic dermatitis may be modulated by multiple biological mechanisms and clinical features.

The significant reduction in peripheral lymphocytes (*P* = 0. 016) confirms immune cells redistribution to AD-affected tissues, a hallmark of AD pathophysiology. This lymphopenia serves as a critical amplifier for NLR and PLR, thereby explaining why NLR (ρ = 0. 045) and PLR (ρ = 0. 050) demonstrate stronger correlations with AD status than individual cell parameters. The synergistic effect of lymphocyte depletion coupled with reactive increases in neutrophils and platelets positions these ratios as sensitive composite biomarkers that integrate both AD-specific adaptive immune responses and secondary systemic inflammation. At the level of immunologic mechanisms, NLR and PLR may be involved in the pathological process of AD through different pathways. First, the inflammatory milieu, characterized by a Th2-type immune response, may promote lymphocyte skin homing through the upregulation of chemokines (e. g., CCL17, CCL22) (5, 6). Concurrently, the activation of the Th17 pathway stimulates neutrophil recruitment (24), a dual mechanism that may explain the predictive value of NLR. Secondly, the activation of platelets leads to the release of PAFs and PMPs, which not only enhance vascular permeability (25) but also promote Th2 differentiation through CD40L-mediated dendritic cell activation (26). This provides a theoretical basis for the association of PLR. Notably, platelet-GPIbα-mediated immunomodulation (27) may form an “inflammatory amplification loop” in patients with asthma co-morbidities, which may explain the strongest association of PLR in this subgroup (OR=1. 84).

The heterogeneity revealed by subgroup analyses warrants further exploration. The observed association appears to be more pronounced in the male population, which may be attributable to the modulatory effects of sex hormones on immune cells. Previous studies have demonstrated that testosterone has been shown to enhance neutrophil activity and suppress Treg function (28). The significant association observed in individuals with a normal BMI suggests the potential interference of metabolic factors with the predictive efficacy of inflammatory markers. Obesity-associated adipokines (e. g., leptin) may mask Th2-type inflammatory features by activating the JAK-STAT pathway (29). The inverse association in nonsmokers may reflect reverse causation. It is plausible that patients with severe dermatitis may have ceased smoking due to heightened health awareness, thereby leading to the confounding of specific clinical features within this subgroup. Additionally, the multifaceted effects of smoking itself on blood cell parameters, such as nicotine suppressing Th17 differentiation, promoting Th2 differentiation and improving Th1/Th2 imbalance (30, 31), may contribute to residual confounding.

This study is subject to several limitations that must be addressed in subsequent research. Firstly, the cross-sectional design precluded the determination of a causal relationship between elevated NLR/PLR and AD, and the possibility of secondary inflammatory responses triggered by skin barrier disruption could not be excluded. Secondly, although multiple covariates were considered, we were unable to rule out the influence of all confounding factors on the results. Furthermore, the diagnosis based on self-reporting may have underestimated the actual prevalence rate, and NHANES lacks objective AD severity measures (e. g., SCORAD), preventing direct NLR/PLR-severity analysis. While pediatric studies have demonstrated a correlation between NLR and severity (15), future cohorts should integrate standardized severity assessments and simultaneous testing of cellular subpopulations (e. g., Th2/Treg ratio) and cytokine profiles, as well as a dynamic monitoring model to assess the temporal relationship between inflammatory markers and disease activity.

## 5 Conclusions

This nationally representative study establishes NLR and PLR as accessible systemic inflammatory biomarkers for AD in US adults. Elevated NLR and PLR were found to be independently associated with 23–24% higher AD prevalence, following comprehensive adjustment for confounders of a sociodemographic and clinical nature. It is imperative to note that we identified significant subgroup heterogeneity. Stronger associations were observed in males, individuals with a normal BMI, and asthmatics. Conversely, paradoxical inverse associations were observed in nonsmokers, which may be indicative of reverse causation or smoking-related immunomodulation. Mechanistically, NLR and PLR integrate dual inflammatory pathways: The condition is characterised by a decrease in the number of lymphocytes in the blood, which is driven by the migration of adaptive immune cells to the skin. Concurrently, there is an increase in the levels of neutrophils and platelets, reflecting Th17 activation and platelet-mediated Th2 amplification. These readily available ratios offer practical tools for risk stratification in high-risk subgroups (e. g., males, asthmatics). It is recommended that future studies adopt longitudinal designs for the purpose of validating their predictive value. In addition, the integration of multi-omics approaches is required in order to facilitate the acquisition of mechanistic insights.

## Data Availability

The datasets presented in this study can be found in online repositories. The names of the repository/repositories and accession number(s) can be found below: https://www.cdc.gov/nchs/nhanes.

## References

[1] Silverberg JI Barbarot S Gadkari A Simpson EL Weidinger S Mina-Osorio P. Atopic dermatitis in the pediatric population: A cross-sectional, international epidemiologic study. Ann Allergy Asthma Immunol. (2021) 126:417–428.e2. doi: 10.1016/j.anai.2020.12.020 33421555

[2] Barbarot S Auziere S Gadkari A Girolomoni G Puig L Simpson EL. Epidemiology of atopic dermatitis in adults: Results from an international survey. Allergy. (2018) 73:1284–93. doi: 10.1111/all.13401 29319189

[3] Simpson CR Newton J Hippisley-Cox J Sheikh A. Trends in the epidemiology and prescribing of medication for eczema in England. J R Soc Med. (2009) 102:108–17. doi: 10.1258/jrsm.2009.080211 PMC2746851 19297652

[4] Langan SM Irvine AD Weidinger S. Atopic dermatitis. Lancet. (2020) 396:345–60. doi: 10.1016/S0140-6736(20)31825-0 32738956

[5] Ferran M Santamaria-Babi LF. Pathological mechanisms of skin homing T cells in atopic dermatitis. World Allergy Organ J. (2010) 3:44–7. doi: 10.1097/WOX.0b013e3181d675f8 PMC3651146 23282416

[6] Czarnowicki T Malajian D Shemer A Fuentes-Duculan J Gonzalez J Suárez-Fariñas M. Skin-homing and systemic T-cell subsets show higher activation in atopic dermatitis versus psoriasis. J Allergy Clin Immunol. (2015) 136:208–11. doi: 10.1016/j.jaci.2015.03.032 25936564

[7] Chan G Wee CP Ong PY. Complete blood count profiles in children with eczema herpeticum. Pediatr Allergy Immunol. (2022) 33:e13648. doi: 10.1111/pai.13648 34379818 PMC8724417

[8] Hu P Liang H Zhao Z Chu H Mo Z Song L. High neutrophil-to-lymphocyte ratio as a cost-effective marker of acute kidney injury and in-hospital mortality after cardiac surgery: a case-control study. Ren Fail. (2024) 46:2417744. doi: 10.1080/0886022X.2024.2417744 39622314 PMC11613334

[9] Hou AC Zhao JY Wei YJ Ou ZH Liu CF. The neutrophil-to-lymphocyte ratio is associated with in-hospital heart failure in patients with ST-segment elevation myocardial infarction treated with primary percutaneous coronary intervention. Heliyon. (2024) 10:e39761. doi: 10.016/j.heliyon.2024 39524828 PMC11550073

[10] Wu Y Chen Y Yang X Chen L Yang Y. Neutrophil-to-lymphocyte ratio (NLR) and platelet-to-lymphocyte ratio (PLR) were associated with disease activity in patients with systemic lupus erythematosus. Int Immunopharmacol. (2016) 36:94–9. doi: 10.1016/j.intimp.2016.04.006 27111516

[11] Liu S Liu J Cheng X Fang D Chen X Ding X. Application value of platelet-to-lymphocyte ratio as a novel indicator in rheumatoid arthritis: A review based on clinical evidence. J Inflammation Res. (2024) 17:7607–17. doi: 10.2147/JIR.S477262 PMC11512772 39464342

[12] Mae Y Takata T Ida A Ogawa M Taniguchi S Yamamoto M. Prognostic value of neutrophil-to-lymphocyte ratio and platelet-to-lymphocyte ratio for renal outcomes in patients with rapidly progressive glomerulonephritis. J Clin Med. (2020) 9:1128. doi: 10.3390/jcm9041128 32326552 PMC7230792

[13] Annen S Horiguchi G Teramukai S Ichiyama S Ito M Hoashi T. Association of transition of laboratory markers with transition of disease activity in psoriasis patients treated with biologics. J Nippon Med Sch. (2022) 89:587–93. doi: 10.1272/jnms.5002 36725002

[14] Hagino T Saeki H Fujimoto E Kanda N. Real-world effectiveness and safety of bimekizumab in Japanese patients with psoriasis: A single-center retrospective study. J Dermatol. (2024) 51:649–58. doi: 10.1111/1346-8138.17186 PMC11484122 38482898

[15] Weissmann S Babyev AS Gordon M Golan-Tripto I Horev A. Hematological markers in children and adults with atopic dermatitis: A retrospective cohort study. Dermatology. (2024) 240:597–605. doi: 10.1159/000539365 38797158

[16] Batmaz SB. Simple markers for systemic inflammation in pediatric atopic dermatitis patients. Indian J Dermatol. (2018) 63:305–10. doi: 10.4103/ijd.IJD_427_17 PMC6052751 30078874

[17] Jiang Y Ma W. Assessment of neutrophil-to-lymphocyte ratio and platelet-to-lymphocyte ratio in atopic dermatitis patients. Med Sci Monit. (2017) 23:1340–6. doi: 10.12659/msm.900212 PMC5367851 28306706

[18] Smith B Collier MR Devjani S Han G Wu JJ. Association between atopic dermatitis and thyroid disease among U. S. adults 2001-2006 Natl Health Nutr Examination Survey. J Am Acad Dermatol. (2023) 88:889–91. doi: 10.1016/j.jaad.2022.10.017 36244551

[19] Iranpour S Sabour S. Inverse association between caffeine intake and depressive symptoms in US adults: data from National Health and Nutrition Examination Survey (NHANES) 2005-2006. Psychiatry Res. (2019) 271:732–9. doi: 10.016/j.psychres.2018.11.004 30791349

[20] Liu J Martin A Thatiparthi A Wu JJ. Association between atopic dermatitis and osteoarthritis among US adults in the 1999-2006 NHANES. J Eur Acad Dermatol Venereol. (2021) 35:e375–7. doi: 10.1111/jdv.17147 33540471

[21] Wei J Jaleel T MacLeod AS Ji JS. Inverted U-shaped relationship between vitamin D and ever-reported eczema in US adults. Allergy. (2019) 74:964–75. doi: 10.1111/all.13708 30589434

[22] Ogdie A Grewal SK Noe MH Shin DB Takeshita J Chiesa Fuxench ZC. Risk of incident liver disease in patients with psoriasis, psoriatic arthritis, and rheumatoid arthritis: A population-based study. J Invest Dermatol. (2018) 138:760–7. doi: 10.1016/j.jid.2017.10.024 PMC6287638 29104161

[23] Inokuchi-Sakata S Ishiuji Y Katsuta M Kharma B Yasuda KI Tominaga M. Role of eosinophil relative count and neutrophil-to-lymphocyte ratio in the assessment of severity of atopic dermatitis. Acta Derm Venereol. (2021) 101:adv00491. doi: 10.2340/00015555-3838 34043019 PMC9413799

[24] Lowes MA Kikuchi T Fuentes-Duculan J Cardinale I Zaba LC Haider AS. Psoriasis vulgaris lesions contain discrete populations of Th1 and Th17 T cells. J Invest Dermatol. (2008) 128:1207–11. doi: 10.1038/sj.jid.5701213 18200064

[25] Ludwig RJ Schultz JE Boehncke WH Podda M Tandi C Krombach F. Activated, not resting, platelets increase leukocyte rolling in murine skin utilizing a distinct set of adhesion molecules. J Invest Dermatol. (2004) 122:830–6. doi: 10.1111/j.0022-202X.2004.22318 15086572

[26] Gisondi P Girolomoni G. Psoriasis and atherothrombotic diseases: disease-specific and non-disease-specific risk factors. Semin Thromb Hemost. (2009) 35:313–24. doi: 10.1055/s-0029-1222610 19452407

[27] Zamora C Cantó E Nieto JC Ortiz MA Diaz-Torné C Diaz-Lopez C. Functional consequences of platelet binding to T lymphocytes in inflammation. J Leukoc Biol. (2013) 94:521–9. doi: 10.1189/jlb.0213074 23801652

[28] Li F Xing X Jin Q Wang XM Dai P Han M. Sex differences orchestrated by androgens at single-cell resolution. Nature. (2024) 629:193–200. doi: 10.1038/s41586-024-07654-z 38600383

[29] Kwa MC Silverberg JI. Association between inflammatory skin disease and cardiovascular and cerebrovascular co-morbidities in US adults: analysis of nationwide inpatient sample data. Am J Clin Dermatol. (2017) 18:813–23. doi: 10.1007/s40257-017-0293-x 28534318

[30] Wu S Zhou Y Liu S Zhang H Luo H Zuo X. Regulatory effect of nicotine on the differentiation of Th1, Th2 and Th17 lymphocyte subsets in patients with rheumatoid arthritis. Eur J Pharmacol. (2018) 831:38–45. doi: 10.1016/j.ejphar.2018.04.028 29715455

[31] Wu S Luo H Xiao X Zhang H Li T Zuo X. Attenuation of collagen induced arthritis via suppression on Th17 response by activating cholinergic anti-inflammatory pathway with nicotine. Eur J Pharmacol. (2014) 735:97–104. doi: 10.1016/j.ejphar.2014 24755145

